# Low-intensity Physical Activity is Associated with Lower Maternal Systemic Inflammation during Late Pregnancy

**DOI:** 10.4172/2165-7904.1000343

**Published:** 2017-06-27

**Authors:** RA Tinius, AG Cahill, WT Cade

**Affiliations:** 1 Program in Physical Therapy, Washington University School of Medicine, USA; 2 Department of Obstetrics and Gynecology, Washington University School of Medicine, USA

**Keywords:** Inflammation, Pregnancy, Physical activity

## Abstract

Excessive maternal inflammation during pregnancy increases the risk for maternal and neonatal metabolic complications. Fortunately, maternal physical activity during pregnancy appears to reduce maternal inflammation. The purpose of this study was to examine the relationship between maternal physical activity intensity and maternal inflammation during late pregnancy. Maternal physical activity levels (sedentary, light, lifestyle, and moderate), fitness levels, and systemic inflammation (plasma C-reactive protein (CRP) concentration) were measured between 32–37 weeks gestation. Relationships were examined by Spearman Rank Coefficient Correlation analyses. Maternal plasma CRP was negatively associated with time spent in light and lifestyle physical activities (Light: r=−0.40, p=0.01; Lifestyle: r=−0.31, p=0.03), but not with time spent in moderate physical activity (r=−0.18, p=0.21). Higher maternal plasma CRP tended to correlate with more time spent sedentary (r=0.27, p=0.06). In addition, increases in light and lifestyle activities may elicit a clinically meaningful change in inflammation. In conclusion, pregnant women should be encouraged to incorporate more low-intensity physical activities into their daily routines in order to decrease systemic inflammation and potentially improve maternal and neonatal pregnancy outcomes.

## Introduction

Low-grade chronic inflammation, often secondary to obesity, plays an important role in the pathogenesis of many chronic diseases including metabolic syndrome, cardiovascular disease, diabetes, and hypertension [1–4]. During pregnancy, maternal systemic inflammation is physiologically elevated [5,6]. Excessive inflammation during pregnancy increases the risk for metabolic complications such as insulin resistance/gestational diabetes and hypertension/preeclampsia [6–8]. Increased inflammation might also increase risk for future maternal disease including metabolic syndrome, insulin resistance, diabetes, hypertension, and cardiovascular disease [9]. Maternal inflammatory changes during pregnancy appear to extend into the placenta potentially exposing the fetus to an inflammatory environment [10], which might contribute to preterm delivery, infection, and the programming of adult metabolic disease [6,11,12].

Light and vigorous maternal physical activities during pregnancy are associated with lower systemic inflammation in the second trimester [13,14]. However, the relationship between physical activity intensity and maternal inflammation in late pregnancy is unclear. The purpose of this study is to examine the relationship between physical activity intensity and inflammation during late pregnancy.

## Materials and Methods

Participants were all part of larger ongoing study cohorts [15,16]. Pre-pregnancy body mass index (BMI) was calculated at initiation of prenatal care. All other measures were taken between 32–37 weeks gestation. Body composition was measured using 7-site skinfold anthropometry (Harpenden Skinfolds Caliper, Baty International, United Kingdom) [17]. Fitness levels were assessed using the YMCA submaximal cycle test on a recumbent bicycle (Lode Corvial Recumbant, Lode B.V., The Netherlands). Maternal physical activity levels were assessed for one week one using the ActiGraph GT3X+ accelerometer (ActiGraph LLC, Pensacola, FL) on the non-dominant wrist. ActiGraph data was collected for seven consecutive days at 30 Hertz (compliance was 100% as all 50 women wore the wristband for all seven days). The percentage of time spent sedentary as well as the amount of time spent participating in different categories of physical activity were calculated using algorithms corresponding to the following activity counts: sedentary: 0–99 counts/min, light: 100–759 counts/min, lifestyle: 760–1951 counts/min, moderate: 1952–5724 counts/min [18]. Inflammation was measured via fasting plasma concentration of high-sensitivity C-reactive protein (CRP).

Spearman’s rank order correlation coefficients were used to assess the degree of relationships between CRP and all other variables. Student’s independent t-tests were used to compare time spent in low-intensity activities between those who are at risk for cardiovascular disease (based on an established cut-off value of CRP ≥ 3.0 mg/L, which represents high-risk for cardiovascular disease [19]) and those who are not (CRP<3.0 mg/L). Logistic regression was also used to determine the amount of physical activity necessary to reduce the odds of having a CRP value ≥ 3.0 mg/L.

## Results

**Figure 1:**
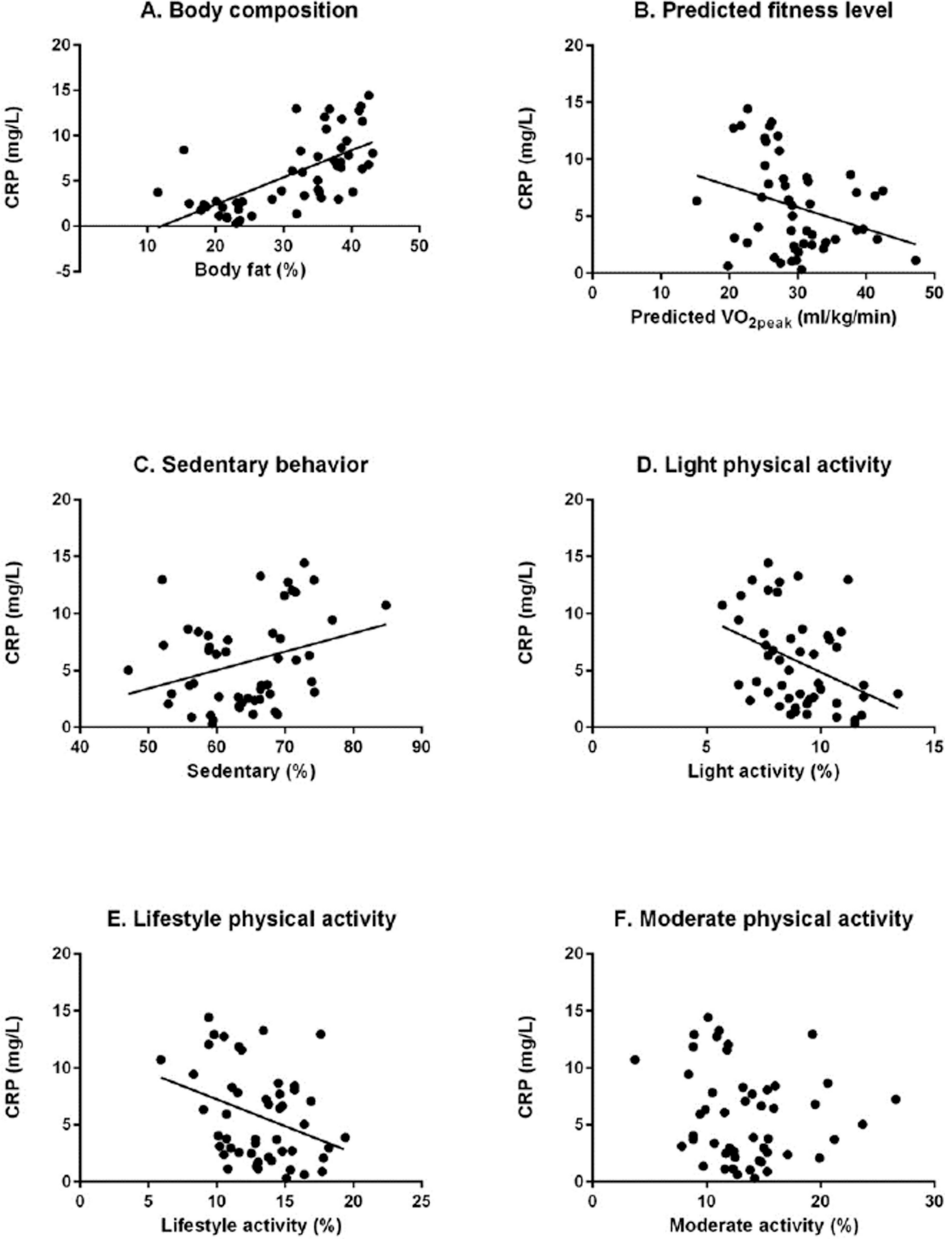
During late pregnancy, maternal inflammation (as measured by CRP) is: (A) positively correlated to maternal body composition; (B) negatively correlated to maternal fitness level; (C) trends towards a positive correlation with sedentary behavior; (D) negatively correlated with light physical activity; (E) negatively correlated with lifestyle physical activity; and (F) has no association to moderate physical activity.

Despite a small sample size, there was an even distribution within the sample among categories of race, parity, and income which might aid in the generalizability of the findings. Maternal plasma CRP concentration was associated with maternal body fat percentage at 32–37 weeks (r=0.70, p<0.001). Higher maternal fitness levels were associated with lower maternal plasma inflammation (r=−0.30, p=0.03). Maternal plasma CRP was negatively associated with time spent in light activity and lifestyle physical activities (light: r=−0.40, p=0.01; lifestyle: r=−0.31, p=0.03). CRP was not correlated with time spent in moderate physical activity (r=−0.18, p=0.21). Higher maternal plasma CRP concentration tended to correlate with more time spent sedentary (r=0.27, p=0.06). Correlations are shown in Figure 1. There were no differences in CRP between the women who only walked and the women who walked in conjunction with other modes of exercise such as biking, swimming, yoga, and weight-lifting (6.2 ± 3.1 mg/L *vs*. 6.3 ± 1.9 mg/L, p=0.94) (Table 1 and Figure 1).

The amount of time spent in low-intensity physical activities (i.e. light or lifestyle) was 24.4 ± 3.8% (351 minutes) for women with a CRP value<3 mg/dl *vs*. 21.1 ± 4.4% (304 minutes) for women who had a CRP value ≥ 3.0 mg/L (p=0.02). The odds of having CRP ≥ 3.0 mg/L were reduced by 16% for every additional 14 minutes per day of low-intensity physical activity (Exp (B)=0.84, 95% CI [0.72–0.98], p=0.03).

## Discussion

The primary finding of our study is that time spent in light and lifestyle physical activities during late pregnancy is associated with lower maternal systemic inflammation. To our knowledge, this is the first study to show relationships between low-intensity maternal physical activity and maternal systemic inflammation during late pregnancy. Understanding the relationships between physical activity intensity and inflammation during late pregnancy (i.e. the present study) is imperative as women are most likely to decrease their physical activity levels and gain additional weight near the end of gestation [20–22]. In addition, our study examined these relationships in a cohort of predominantly obese women, which is important because these women are at the highest risk for excessive maternal inflammation and its downstream sequela [23,24].

Additionally, time spent sedentary during pregnancy tended to be associated with higher maternal systemic inflammation. Surprisingly, there was not a significant relationship between time spent in moderate physical activity and inflammation. These data suggest that low-intensity physical activity may reduce inflammation in pregnant women and that decreasing time spent sedentary and increasing low-intensity activities may be enough of a stimulus to elicit reductions in inflammation. Our findings also suggest that more intense physical activities might not necessarily be superior to walking during late pregnancy in terms of lowering maternal inflammation; however more research in necessary to confirm this.

Upon further analysis, our data suggest that spending an additional 14 minutes per day in light or lifestyle physical activities may reduce the odds of having a CRP value over the clinical threshold of 3.0 mg/L by 16%; however, this should be confirmed in a larger cohort. In addition, pregnant women with CRP values below this clinical threshold spent on average only 47 additional minutes per day doing light and lifestyle activities than those who were above this clinical value. Therefore, even small daily increases in light and lifestyle activities could elicit a clinically meaningful change in inflammation.

Based on the present study, we believe pregnant women should be encouraged to increase physical activity during day-to-day tasks (e.g. taking the stairs, parking further away, walking instead of driving) in order to reduce systemic inflammation late in pregnancy. Not only do these types of lifestyle changes appear to elicit reductions in maternal systemic inflammation, but they are easier for pregnant women to adopt compared to structured exercise programs. In fact, the most commonly reported barriers to physical activity during pregnancy are lack of time, employment obligations, childcare responsibilities, fatigue, and pregnancy-related discomfort [25–29]. Incorporating less sedentary time and more low-intensity physical activities into daily routines might help women overcome these barriers as these modifications do not involve large time commitments (e.g. does not require a blocked-off period of time to go the gym); they can be performed in the workplace (e.g. take the stairs at work, use a standing desk, take frequent breaks at work to walk); they can be done with children (e.g. playing in the yard with other kids or pets, going for a slow walk with a stroller or wagon, walking around a store); and they are not overly fatiguing activities that may be uncomfortable during late pregnancy.

## Figures and Tables

**Table 1:** Maternal demographic and metabolic characteristics (N=50).

Characteristics	mean ± SD

Age (year)	26.6 ± 5.0

Pre-pregnancy BMI (kg/m^2^)	30.2 ± 7.5

Body fat percentage (%)	31.2 ± 8.8

Resting systolic blood pressure (mmHg)	110.2 ± 9.4

Resting diastolic blood pressure (mmHg)	70.0 ± 6.8

Resting heart rate (bpm)	87.4 ± 10.8

Gestational weight gain (kg)	11.8 ± 7.1

C-Reactive protein (mg/L)	5.7 ± 4.0

Race	

African-American	26 (52%)
Caucasian	21 (42%)
Other	3 (6%)

Parity	

Nulliparous	28 (56%)
Multiparous	22 (44%)

Income	

Low	23 (46%)
Moderate-to-high	27 (54%)

Physical activity levels (%/week)	

Sedentary	64.3 ± 7.5
Light	9.1 ± 1.7
Lifestyle	13.1 ± 2.9
Moderate	13.5 ± 4.3

Predicted VO_2max_ (ml/kg/min)	29.7 ± 6.4

Data are presented as mean ± SD for continuous variables and number of women (%) for discrete variables.

## Take-home points

- Maternal inflammation during late pregnancy is negatively correlated with low-intensity physical activities (light, lifestyle) but not moderate intensity activities. Thus, low-intensity activities may help lower systemic inflammation during late pregnancy.
- Spending less total time in sedentary may be enough of a stimulus to reduce inflammation during late pregnancy.
- Even small increases in the duration of time spent in low-intensity physical activities during late pregnancy may elicit clinically meaningful reductions in CRP.
- Pregnant women of all body weights should be encouraged by their health care providers to find low-intensity activities that are enjoyable and can be easily incorporated into their daily lives.
